# Intensity modulated radiotherapy (IMRT) in benign giant cell tumors -- a single institution case series and a short review of the literature

**DOI:** 10.1186/1748-717X-5-18

**Published:** 2010-02-26

**Authors:** Falk Roeder, Carmen Timke, Felix Zwicker, Christian Thieke, Marc Bischof, Jürgen Debus, Peter E Huber

**Affiliations:** 1 Clinical Cooperation Unit Radiation Oncology, German Cancer Research Center (DKFZ), Heidelberg, Germany; 2 Department of Radiation Oncology, University of Heidelberg, Heidelberg, Germany

## Abstract

**Background:**

Giant cell tumors are rare neoplasms, representing less than 5% of all bone tumors. The vast majority of giant cell tumors occurs in extremity sites and is treated by surgery alone. However, a small percentage occurs in pelvis, spine or skull bones, where complete resection is challenging. Radiation therapy seems to be an option in these patients, despite the lack of a generally accepted dose or fractionation concept. Here we present a series of five cases treated with high dose IMRT.

**Patients and Methods:**

From 2000 and 2006 a total of five patients with histologically proven benign giant cell tumors have been treated with IMRT in our institution. Two patients were male, three female, and median age was 30 years (range 20 -- 60). The tumor was located in the sacral region in four and in the sphenoid sinus in one patient. All patients had measurable gross disease prior to radiotherapy with a median size of 9 cm. All patients were treated with IMRT to a median total dose of 64 Gy (range 57.6 Gy to 66 Gy) in conventional fractionation.

**Results:**

Median follow up was 46 months ranging from 30 to 107 months. Overall survival was 100%. One patient developed local disease progression three months after radiotherapy and needed extensive surgical salvage. The remaining four patients have been locally controlled, resulting in a local control rate of 80%. We found no substantial tumor shrinkage after radiotherapy but in two patients morphological signs of extensive tumor necrosis were present on MRI scans. Decline of pain and/or neurological symptoms were seen in all four locally controlled patients. The patient who needed surgical salvage showed markedly reduced pain but developed functional deficits of bladder, rectum and lower extremity due to surgery. No severe acute or late toxicities attributable to radiation therapy were observed so far.

**Conclusion:**

IMRT is a feasible option in giant cells tumors not amendable to complete surgical removal. In our case series local control was achieved in four out of five patients with marked symptom relief in the majority of cases. No severe toxicity was observed.

## Background

Giant cell tumors of bone are usually benign tumors, however they can be locally aggressive and in some cases malignant transformation or metastatic disease occurs [1,2]. They account for approximately 5% of all primary bone tumors and about 20% of benign bone tumors [1]. The majority of these tumors is located in the long bones of the extremities, however a small proportion (< 10%) occurs in the pelvis, spine or skull base [1,2]. Usually patients present with small lesions after a brief history of swelling or pain but especially in the sacral region, giant cell tumors can reach an enormous size and result in massive pain in combination with severe neurological deficits. The standard of care for giant cell tumors is function-preserving surgery [3]. After complete resection, local control is achieved in 85-90% of all cases [3], but incomplete resection is frequently associated with tumor recurrence in up to 50% of the cases [4]. Despite the improvements in surgical techniques, complete tumor removal without major functional deficits remains challenging in some regions, especially sacral or pelvic bones, spine or skull base [4]. Therefore primary radiotherapy has been advocated as an alternative treatment in patients suffering from giant cell tumors in these regions, although concerns about local side effects of radiotherapy with appropriate doses have been raised in the past [5,6]. As radiotherapy techniques have extensively evolved in the last decades, including the development of three-dimensional conformal radiotherapy with megavoltage energies and even intensity-modulated and image-guided radiotherapy, the possibility to apply high doses with less toxicity and optimal sparing of critical structures is now widely available. Here we report our experience with intensity-modulated radiotherapy in the treatment of giant cell tumors occurring outside the extremities in combination with a short review of the literature.

## Patients and Methods

Between 2000 and 2006 a total of five patients with giant cell tumors have been treated with intensity modulated radiotherapy in our institution. All tumors were histologically proven before start of the treatment. All patients except one with a giant cell tumor in the sphenoid sinus suffered from large tumors in the sacral region. Three tumors were judged primarily irresectable, and one patient had undergone a subtotal resection prior to radiotherapy. One patient suffered from a local recurrence after initial surgery and embolisation and received another embolisation and a subtotal resection of the recurrence before irradiation. All patients with tumors in the sacral region suffered from massive pain and sensory neurological deficits prior to radiotherapy. For detailed patient characteristics see table 1.

**Table 1:** Patients, treatment and outcome

Pat.	Age	Gender	Local.	Size	Treatm.	Dose	f/u	Local Recurrence	Clinical Outcome	Radiographic Outcome
1	60	F	Sacral	3,5	E+S*+RT	64	107	No	Minor improvement	No change
2	52	F	Sacral	9	RT	64	46	3 months (salvage)	Progressive symptoms	No change
3	23	M	Sphenoid	2,5	S*+RT	57,6	63	No	No residual symptoms	No change
4	20	M	Sacral	10	RT	66	44	No	Major improvement	Tumor necrosis
5	30	M	Sacral	11	RT	60	35	No	Major improvement	Tumor necrosis

age [years], M: male, F: female, size [cm], S*: surgery (subtotal resection), E: embolisation, RT: radiotherapy, dose [Gy], f/u: follow up [months]

**Figure 1:**
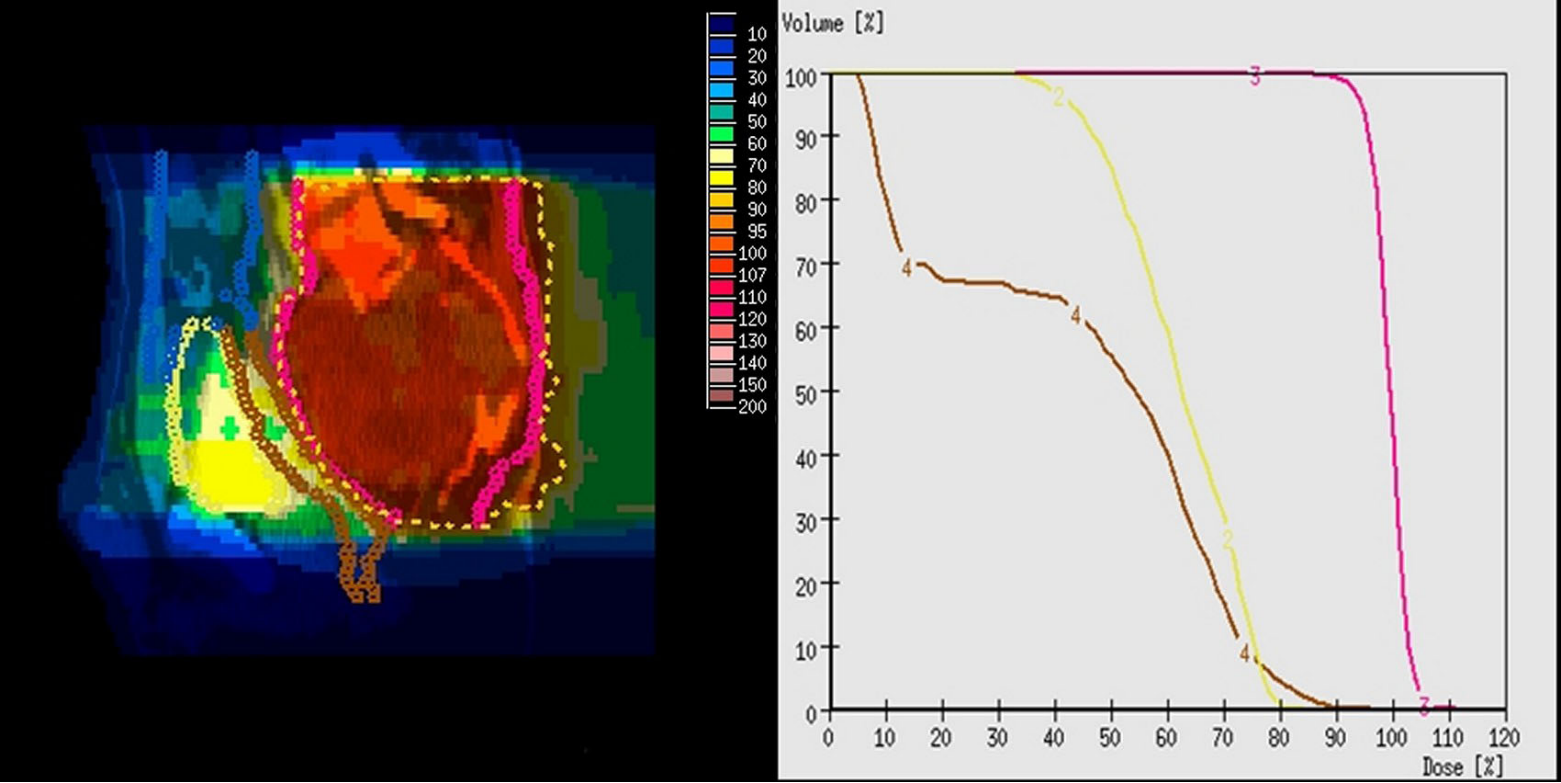
**Sagittal dose distribution and DVH information in patient 5**. graphs: PTV (3), rectum (4), bladder (5)

**Figure 2:**
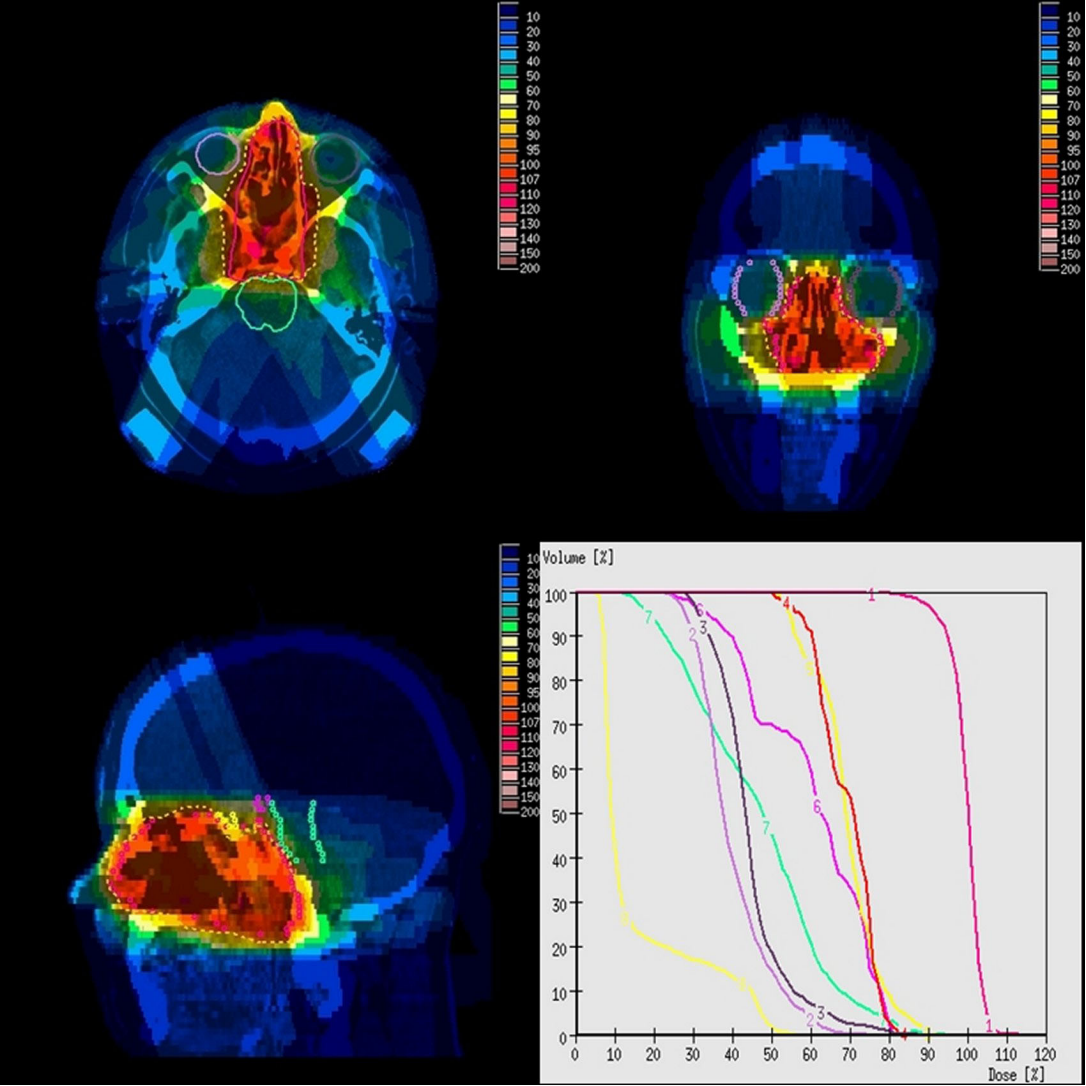
**Transversal, coronar and sagittal dose distribution and DVH information in patient 3**. graphs: PTV (1), left eye (2), right eye (3), right optic nerve (4), left optic nerve (5), chiasma (6), brainstem (7), spinal cord (8)

All patients were treated with IMRT using the step-and-shoot approach [7]. For treatment planning, patients were fixed in an individually manufactured precision head and body mask made of Scotch cast^® ^(3 M, St.Paul, Minneapolis, MN) or an individually fixed vacuum pillow in order to immobilize the body. With this immobilization system attached to the stereotactic base frame, we performed contrast-enhanced CT- and MRI-images under stereotactic conditions, with a slice thickness of 3 mm. We scanned the whole treatment region with a superior and inferior margin of at least 3 cm. After stereotactic image fusion based on the localizer-derived coordinate system [8,9], all critical structures as well as the target volumes were defined on each slice of the three-dimensional data cube. The gross tumor volume (GTV) was defined as the macroscopic tumor visible on CT- and MRI-scans. For the clinical target volume (CTV) a margin of 1-2 cm was added. In cases of subtotal resections the whole resection cavity was included into the CTV. Inverse treatment-planning was performed using the KonRad software developed at the German Cancer Research Center (DKFZ), which is connected to the 3D planning program VIRTUOS to calculate and visualize the 3D dose distribution. The IMRT treatment planning process has been described in detail previously [10-13]. Radiation treatment was delivered by a Siemens accelerator (Primus, Siemens, Erlangen, Germany) with 6 or 15 MV photons using an integrated motorized multileaf collimator (MLC) for the step-and-shoot technique automatically delivering the sequences. The total doses were prescribed to the median of the target volume and usually the 95% isodose surrounded the CTV. The prescribed dose ranged from 57.6 Gy to 66 Gy with a median dose of 64 Gy, applied in conventional fractionation (single dose 1.8-2 Gy, five fractions per week). Examples for dose distributions and DVH data are shown in Figure 1 and 2. Time to event data was calculated from the first day of radiation treatment. Local progression was defined as tumor growth on repeated CT or MRI scans or increase of clinical symptoms which needed surgical salvage.

## Results

All patients were followed with clinical examination and MRI scans in our institution or the referring hospital on a regular basis. Median follow up was 46 months, ranging from 30 to 107 months.

### Local control and salvage surgery

Four out of five patients have been locally controlled without clinical or radiographic signs of progression, resulting in an overall local control rate of 80%. One patient with a biopsy proven primary giant cell tumor of the sacral region developed a progression of clinical symptoms in the meaning of pain, paralysis of the leg and bladder/rectal dysfunction without tumor progression on MRI scan three months after radiotherapy. She received salvage surgery which included complete removal of the tumor and is currently alive without evidence of disease and marked pain relief, but suffers from impaired extremity function, complete loss of bladder function and a permanent descendostoma.

### Treatment toxicity

Acute toxicity related to the radiation treatment was of minor grade in all cases. No acute toxicity of grade > 1 according to RTOG was observed. In detail, three patients suffered from mild skin erythema, one from mild alopecia, one from diarrhea, one from urgency and one from mild conjunctivitis. All acute toxicities resolved spontaneously. Beside from mild skin hyperpigmentation in the irradiated areas in two patients, no late toxicities attributable to radiation therapy were observed so far.

### Clinical outcome

Reduction of pain was observed in four out of five patients already during radiotherapy. Considering the long term follow up excluding the patient with salvage surgery three months after radiotherapy, one patient showed a minor, two patients a major improvement of their symptoms and one patient is free of symptoms. Improvement included not only reduced pain but also a decrease of the sensory neurological deficits in two patients.

### Radiographic outcome

**Figure 3:**
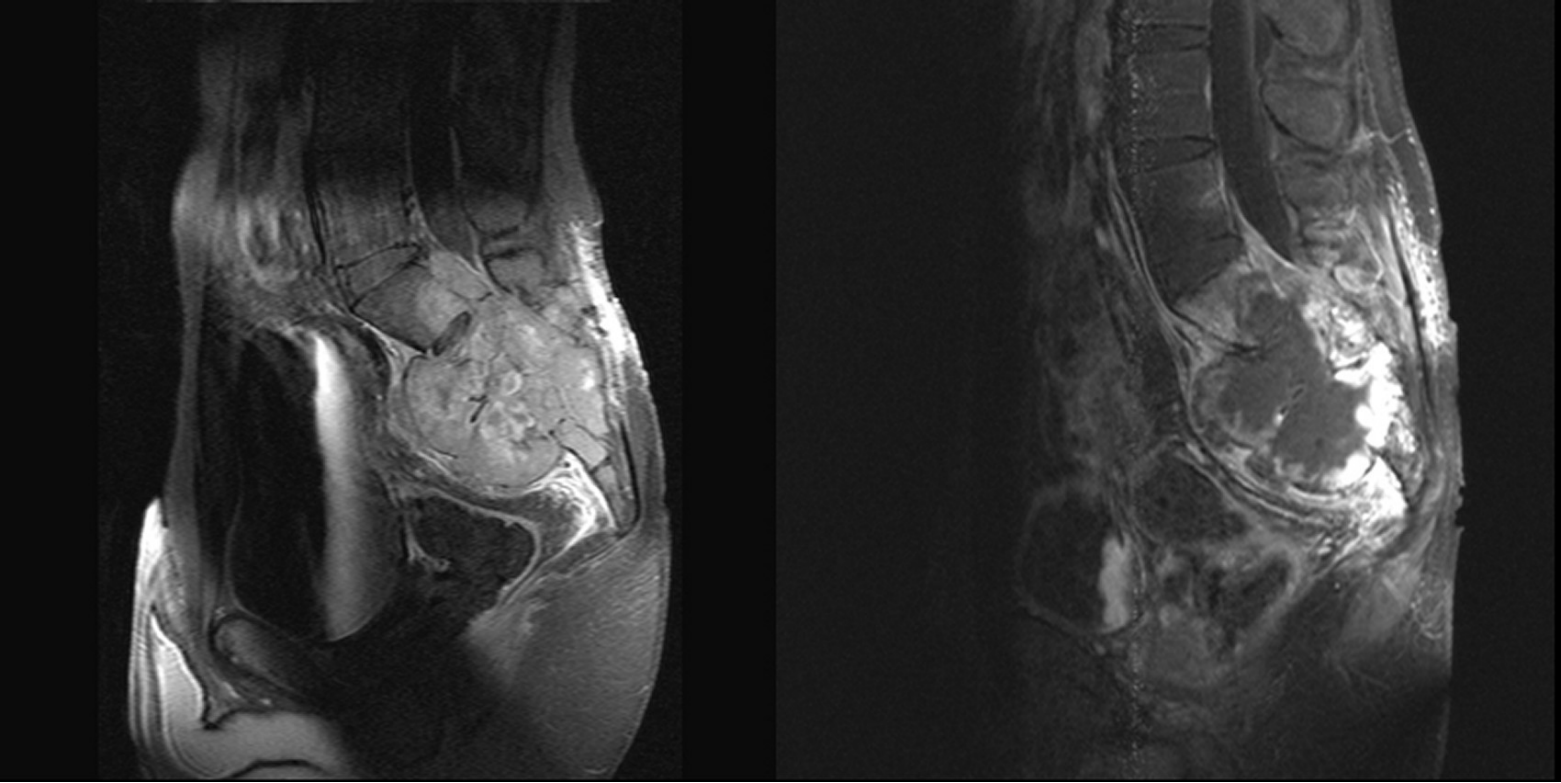
**Development of central tumor necrosis in patient 4**. left side: MRI before radiotherapy, right side: MRI 1 year after radiotherapy with development of massive central necrosis

All patients were monitored closely with repeated MRI imaging during the follow up period. None of the patients showed a substantial reduction of tumor size after radiotherapy, but in two patients typical radiographic signs of massive central tumor necrosis were found as reaction on radiotherapy during the further follow up (see figure 3).

## Discussion

The mainstay of treatment of giant cell tumors of the bone is complete surgical excision. Especially in patients with extremity tumors, this treatment results in high local control rates of more than 85% [3] without major complications or functional deficits. However, a small proportion of patients suffers from large giant cell tumors of sacral bone, spine or skull base. In these regions of the body, complete surgical removal without major functional deficits is challenging or sometimes impossible and recurrence rates of about 50% have been reported after surgical treatment with intralesional margins [4]. Systemic treatment options are limited, although there seems to be some progress through improved understanding of the molecular mechanisms in the development of giant cell tumors. As they are rich in stromal cells that express RANKL, a key mediator of osteoclast activation [14], increasing interest has been paid to monoclonal antibodies against RANKL, for example denosumab. A pilot study in 37 patients showed a response rate of 86% and functional improvements including reduced pain in 84% of the patients suffering from giant cell tumors treated with denosumab [14]. However, no long term data about the recurrence rate, functional outcome and long term toxicity with this promising approach exists so far and therefore further investigation is needed to establish the value of this treatment option. Therefore primary radiotherapy has to be considered as an alternative treatment in patients with giant cell tumors not suitable for complete resection, although based on small patient series, collected over long time periods, with wide variations in fractionation, total dose and radiation techniques [1-4,15-20].

Beside the limited data for this treatment approach, radiotherapy has been criticized in the past also because of low rates of local control in some series and concerns about side effects and induction of malignant transformation [2,5,6]. Careful examination reveals that many of these series have been conducted in the 2-D era of radiotherapy and radiodiagnostics more than 15 years ago. This implicates not only a high possibility for geographical misses due to the use of plain radiographs for tumor localisation, which could have resulted in decreased coverage of the tumors by radiation therapy and therefore decreased local control, but also the use of orthovoltage techniques with low energies, resulting in high toxicity due to the unfavourable dose distribution and probably increased rates of secondary malignancies [21].

As radiation therapy techniques have strongly improved in the last decades including the wide-spread implementation of three-dimensional conformal radiotherapy and even intensity-modulated and image-guided radiotherapy, these lesions can now be treated with high doses in the absence of major acute and late side effects to the adjacent normal tissues. In our case series, five patients were treated with intensity modulated radiotherapy to a median dose of 64 Gy, which resulted in a local control rate of 80%. Although all primary tumors have been localized in regions with directly adjacent organs at risk, like rectum, small bowel or the optic nervous system, no severe acute or late toxicity attributable to radiation treatment has been observed so far. Other series using modern radiation therapy techniques have reported similar results. For example Feigenberg et al. [1] found a local control rate of 77% in a series of 26 lesions with three severe and four minor complications associated with radiotherapy using doses of 35-55 Gy. Schwartz et al. [15] reviewed the MGH experience and observed a local control rate of 85% after radiotherapy with doses of 42-68 Gy. Seider at al. [3] presented a series from the MD Anderson and found a local control rate of 70% using doses of 36-66 Gy. Even after exclusion of all non-extremity tumors and all patients with gross total resection prior to radiotherapy from these series, the results do not differ distinctly (see table 2). Thus modern imaging and radiation techniques offer the possibility of high tumor control rates without major side effects.

**Table 2:** Literature overview

Author	Year	n	f/u^8^	Size^9^	RT dose^10^	LR
Seider et al.[3]	1986	10	8	n.s.	45,5	30%
						
Schwartz et al.[15]^1,2^	1989	7	4	7	54	14%
						
Malone et al. [2]^1,2^	1995	5	19	7,5	35^7^	20%
						
Feigenberg et al. [1]^1,2^	2003	15	10	n.s.	45	20%
						
Leggon et al. [4]^1,3^	2004	11	6	10	47,8	18%
						
Leggon et al. [4]^1,3,4^	2004	148	9^6^	n.s.	47,8^5^	47%
						
Own data	2009	5	4	9	64	20%
						

Selected reports dealing with non-extremity giant cell tumors treated with RT alone or after subtotal resection, ^1 ^: only patients with macroscopic residual disease after surgery or primary treatment included, ^2^: only patients suffering from non-extremity lesions included, ^3 ^: only patients treated with RT included, ^4 ^: pooled literature analysis, ^5 ^: mean dose, ^6 ^: mean f/u calculated of the entire cohort including patients without RT, ^7 ^: single dose 2,4 Gy, ^8 ^: [years], ^9 ^: [cm], ^10 ^: median dose [Gy], LR: crude local failure rates, f/u: median follow up

Considering the issue of malignant transformation, these concerns regarding radiation therapy, have mainly been based on initial reports of transformation rates up to 24% [6]. Other series using more modern radiotherapy techniques found lower rates of 0-11% [1,4] and a recent metaanalysis reported an incidence of less than 1% in patients treated with megavoltage radiation and modern radiation therapy techniques [1]. Beside that, malignant transformation and sarcoma induction have also been reported in patients treated without radiation at all. For example Dahlin et al. [22] reported the development of sarcoma in 2 of 47 (4%) patients and Mnaymneh et al. [23] even in 2 of 25 (8%) patients after surgery. The appearance of malignant giant cell tumors of bone or malignant foci inside benign giant cell tumors has been described also in a small number of patients [24,25], and pulmonary metastases can be found in 2-9% of patients with benign giant cell tumors [5,26-28]. Thus malignant transformation or the appearance of metastases could be part of the disease itself in a small proportion of cases and should not be attributed unreflected to radiation treatment.

To date there is no generally accepted fractionation or dose concept for the treatment of giant cell tumors. A clear dose-effect relationship has not been established yet, but in some series higher doses resulted in increased local control rates. For example, Feigenberg et al. [1] found a significant increased local control rate of 86% with doses above 40 Gy compared to 67% with lower doses. In contrast, Leggon et al. [4] did not find a benefit in terms of local control comparing doses of < 45 Gy, 45-55 Gy and > 55 Gy in pelvic and sacral lesions, but the overall local control rate in their series was only about 50%. Malone et al. [2] found a local control rate of 83% in non-extremity lesions even using doses as low as 35 Gy in 15 fractions. In our patients, we attempted doses of 60-66 Gy, a dose range which could be safely administered without major toxicities based on our experiences in treating other sacral lesions like chordoma or low grade chondrosarcoma using IMRT in order to achieve maximal local control. Although a wide dose range was reported in most of the series, careful examination leads to the impression that usually patients with radiation as sole treatment and non-extremity lesions were treated with higher doses. However, if dose escalation beyond doses of 45 Gy increases local control, remains an open question based on the available data.

Considering the clinical outcome of patients with giant cell tumors treated by radiotherapy, only little information is available in the literature. For example in the series of Schwartz et al. [15], only three of thirteen patients had neurological symptoms before treatment. All three patients showed improved neurological function after radiation therapy. Malone et al. [2] reported 7 patients with symptomatic disease before radiotherapy, all have been ambulatory and independent after treatment. In our series, all patients suffered from pain and/or neurological deficits prior to radiotherapy. After treatment, all patients showed some kind of improvement except the patient who needed salvage surgery three months after radiotherapy. One of the four patients is free of symptoms, two had major improvements and one a minor improvement. Thus radiotherapy cannot only stop the locally destructive growth of giant cell tumors but also decreases pain and other neurological symptoms of the patients resulting in improved quality of life.

Considering the radiographic outcome of giant cell tumors after radiotherapy, the available information in the literature is even more scanty than for clinical outcome. This may be linked to the use of two-dimensional radiographs for diagnosis and follow up in most of the older series. The appearance of bone sclerosis after radiotherapy in most cases has been described by Seider et al. [3], and tumor response in terms of involution or ossification was observed in 4 of 9 patients in the series reported by Leggon et al. [4]. In our series, MRI was used for diagnostics and regular follow up in all patients. In contrast to the mentioned results, we did not find significant tumor volume shrinkage after treatment. However, the absence of significant volume reduction is a common feature of benign lesions treated by radiotherapy, as shown in many other entities like meningioma, desmoids or chordoma [29-31] and should not be interpreted as a failure of treatment.

## Conclusion

Radiotherapy carried out by modern techniques based on modern imaging could be an alternative treatment approach in patients with giant cell tumors not amendable to function-preserving surgery. High local control rates without severe acute or late side effects and improvement in clinical symptoms are achievable in the majority of patients.

## Competing interests

The authors declare that they have no competing interests.

## Authors' contributions

FR participated in data acquisition, literature review and drafted the manuscript. CTI, FZ and CTH participated in data acquisition and literature review. MB, JD and PEH participated in drafting the manuscript and revised it critically. All authors read and approved the final manuscript.
